# Pharmacological Evaluation of *Acacia nilotica* Flower Extract against *Helicobacter pylori* and Human Hepatocellular Carcinoma In Vitro and In Silico

**DOI:** 10.3390/jfb14040237

**Published:** 2023-04-21

**Authors:** Aisha M. H. Al-Rajhi, Husam Qanash, Abdulrahman S. Bazaid, Naif K. Binsaleh, Tarek M. Abdelghany

**Affiliations:** 1 Department of Biology, College of Science, Princess Nourah bint Abdulrahman University, P.O. Box 84428, Riyadh 11671, Saudi Arabia; amoalrajhi@pnu.edu.sa; 2 Department of Medical Laboratory Science, College of Applied Medical Sciences, University of Ha’il, P.O. Box 84428, Hail 55476, Saudi Arabia; h.qanash@uoh.edu.sa (H.Q.); ar.bazaid@uoh.edu.sa (A.S.B.); n.binsaleh@uoh.edu.sa (N.K.B.); 3 Botany and Microbiology Department, Faculty of Science, Al-Azhar University, Cairo 11725, Egypt

**Keywords:** *Acacia nilotica*, flowers, *Helicobacter pylori*, hepatocellular carcinoma, in silico

## Abstract

The resistance of cancer and *Helicobacter pylori* to several drugs reflects a worldwide problem, and it has been the intention of numerous researchers to overcome this problem. Thus, in this study, *Acacia nilotica* fruits were subjected to HPLC analysis to detect their phenolic compounds and flavonoids. Moreover, *A. nilotica*‘s anti-*H. pylori* activity and its inhibitory activity against human hepatocellular carcinoma (HepG-2 cells) were reported. Various compounds with different concentrations, such as ferulic acid (5451.04 µg/mL), chlorogenic acid (4572.26 µg/mL), quercetin (3733.37 µg/mL), rutin (2393.13 µg/mL), gallic acid (2116.77 µg/mL), cinnamic acid (69.72 µg/mL), hesperetin (121.39 µg/mL) and methyl gallate (140.45 µg/mL), were detected. Strong anti-*H. pylori* activity at 31 mm was reported, compared to the positive control of the 21.67 mm inhibition zone. Moreover, the MIC and MBC were 7.8 µg/mL and 15.62 µg/mL, respectively, while the MIC and MBC of the positive control were 31.25 µg/mL. The concentration of MBC at 25%, 50% and 75% reflected *H. pylori*’s anti-biofilm activity of 70.38%, 82.29% and 94.22%, respectively. Good antioxidant properties of the *A. nilotica* flower extract were documented at 15.63, 62.50, 250 and 1000 µg/mL, causing the DPPH scavenging percentages of 42.3%, 52.6%, 65.5% and 80.6%, respectively, with a IC_50_ of 36.74 µg/mL. HepG-2 cell proliferation was inhibited (91.26%) using 500 µg/mL of flower extract with an IC_50_ of 176.15 µg/mL, compared to an IC_50_ of 395.30 µg/mL used against human normal melanocytes. Molecular docking was applied to investigate ferulic acid with the *H. pylori* (4HI0) crystal structure to determine the best binding mode that interacted most energetically with the binding sites. Molecular docking indicated that ferulic acid was a proper inhibitor for the 4HI0 protein enzyme of *H. pylori*. A low energy score (−5.58 Kcal/mol) was recorded as a result of the interaction of ferulic acid with the residue’s SER 139 active site caused by the O 29 atom, which was important for its antibacterial activity.

## 1. Introduction

Since the dawn of ancient times, humans have used medicinal plants, and they have continued to play a significant role in the development of safer and more efficient natural drug delivery methods [1,2,3]. The black babul, kikar or the gum arabic tree, often referred to as *Acacia nilotica* (native to Egypt), is a member of the Fabaceae family, which is extensively distributed throughout several nations. Being a common and significant plant, it is used in a variety of ways from medicine (root, bark, leaves, forbear, gum and pods) to feed (leaves and shoots for animals) and dyeing (leather color) [4]. All parts of *A. nilotica*, including the seeds, gum, flowers, leaves, bark, roots and pods, are therapeutically important and have been applied in the traditional medicines [5]. It is effective in the treatment of coughs, congestion, cold, nerve stimulation, dysentery, leucorrhea, hemorrhages, and ophthalmia, can offer sclerosis relief and wound healing, and has antiulcer, anti-inflammatory, anthelmintic, diuretic, antihypertensive and antipyretic effects [6]. Pharmacological reports have indicated that *A. nilotica* extract has antioxidant activity and insulin sensitizing properties, which help with declining obesity rates and minimizing hyperlipidemia [7]. Edible and nutritional characteristics were associated with *A. nilotica* seed due to the presence of numerous phytochemicals [8].

Using a variety of in vitro experiments, Kaur et al. [4] extracted betulin from the ethyl acetate fraction of the methanol extract of *A. nilotica* and evaluated its various antioxidant, cytoprotective and anti-inflammatory properties. *A. nilotica* may be found in many tropical and subtropical areas. It has been used to prevent and treat a variety of illnesses and infectious disorders since it contains many bioactive chemicals. Extracts of *A. nilotica* have positive antioxidant and antimalarial properties [9]. The plant contains many polyphenolic chemicals such as catechins, which are thought to have anti-inflammatory and antioxidant properties [10]. Hepatitis C virus protease and multidrug resistant bacterial pathogens have both been shown to be inhibited by *A. nilotica* [11]. Several phenolic constituents with an extensive range of biological activity were found in the plant’s aerial parts [12]. The use of green extraction techniques has developed and increased in recent years to obtain the natural molecules. These techniques, such as enzyme-aided extraction, pressurized liquid extraction, supercritical fluid extraction, ultrasonic/microwave-assisted extraction, maceration and hydrodistillation, have been used to increase the yield of extracted molecules and reduce the negative impact of chemicals on the environment [13,14,15]. The biological effects of flower and leaf extracts from *Acacia* species, notably, *A. saligna,* have not been well studied. The antioxidant and antibacterial effects of Egyptian *A. saligna* flower water extracts were ineffective, while the antifungal effects were strong [16]. In comparison to *A. laeta* extracts, Egyptian *A. nilotica* and *A. seyal* leaf extracts have shown stronger antioxidant activity [17]. Previous studies have demonstrated that leaf extracts of the Egyptian *A. saligna* may qualitatively include phenolic acids (e.g., gallic acid) and flavonoids (e.g., quercetin, quercitrin, apigenin, apigenin 7-glucoside, astragalin, luteolin, myricetin and kaempferol) [18]. A study conducted in an eastern region of Saudi Arabia on the aerial parts of *Acacia* species (*A. salicina, A. laeta, A. hamulosa* and *A. tortilis*) showed significant cytotoxic activity in *A. laeta* and *A. hamulosa* against HepG2 and breast cancer cell lines [19]. *Hordeum murinum* was resistant to flower extracts from other *Acacia* species [20]. Another study on the flower’s extracts has documented the presence of phenolic acids (benzoic acid, caffeic acid, o-coumaric acid, *p*- hydroxybenzoic acid and ellagic acid) and flavonoids (quercetin, naringenin and kaempferol) [16].

It is known that *Helicobacter pylori*, which thrives and multiplies in acidic environments, is typically responsible for gastric and duodenal ulcers [21,22]. It is well known that, early in life, human stomachs can suffer from the invasion of *H. pylori*, but the detection of this pathogen may be discovered later. Although 50% of individuals worldwide are infected with *H. pylori*, as mentioned in several reports from developing nations [20,21], but about 15% are only characterized with the appearance of infection symptoms, such as gastritis, peptic ulcer and gastric adenocarcinoma [23]. *H. pylori* infection was induced by urease (a nickel-containing enzyme) via an appropriate pH to prolong its survival; thus, the urease activity inhibition should be a strong tool to control the infection of *H. pylori*. The urease of *H. pylori* breaks down urea into ammonia, and the released ammonia shields it from the stomach’s acidic environment.

Until now, the management of *H. pylori* infections is a worldwide problem, so the discovery or development of bioproducts can represent a strategy to overcome this problem. An *A. vera* gel loaded with chitosan nanoparticles was a promising treatment tool for *H. pylori* infection [24], as documented in the minimum inhibitory concentration (MIC) and minimum bactericidal concentration (MBC) of the *A. nilotica* leaf extract against *H. pylori* of 180 g/mL and 5 mg/mL, respectively. The stronger anti-*H. pylori* and significant inhibition of urease activity has been reported via the extracts of *A. nilotica* [25]. In vitro and in vivo tests of the *A. nilotica* extract revealed high anti-urease activity and significant anti-adhesion properties [26]. Therefore, these studies indicated that *A. nilotica* could influence the colonization of *H. pylori* in the stomach, and its potential pharmaceutical use for the eradication of *H. pylori* could be further investigated. Moreover, these compounds were chosen for molecular docking studies with targeted *H. pylori* virulent proteins such as cytotoxic-associated gene A (CagA), vacuolating-associated gene (VacA), urease, and gastric cancer-induced signaling proteins such as the epidermal growth factor receptor and the vascular endothelial growth factor receptor. All the compounds of the *A. nilotica* leaf extract demonstrated good drug-likeness scores. *A. nilotica* leaf extract could act as a potent anti-*H. pylori* and a gastric cancer drug, as demonstrated by the validation of the in vitro and in silico results of Sampath et al. [27].

Different species of *Acacia* were tested to repress the proliferation of several cancer cells. For instance, kidney, breast and liver cells were repressed by the extract of aerial parts of *Acacia hamulosa*, *A. salicina*, *A. laeta* and *A. tortilis* [19]. In addition, human cancer cell lines, such as those of MCF-7, A549 and THP-1 cells, were influenced by *A. catechu* and *A. nilotica* extracts [28,29]. *A. nilotica* leaf extracts exhibited anticancer activity against Hep-2 (cervical cancer) and MDA-MB-231 (breast cancer) [30]. Numerous scientific texts have almost wholly focused on the biological screening as well as the phytochemical constituents of the leaves, seeds, pod, root and bark of *A. nilotica*. However, the biological effects of *A. nilotica* flowers are limited. In this study, therefore, an investigation has been conducted to test the extract of *A. nilotica* flowers against *H. pylori* and its anticancer activities.

## 2. Materials and Methods

### 2.1. Chemicals 

Analytical grade chemicals were obtained from Sigma-Aldrich, Taufkirchen, Germany, including DPPH (2,20 -diphenyl-1-picrylhy drazyl), dimethyl sulfoxide (DMSO), ascorbic acid, trypan blue dye, crystal violet, L-glutamine, 25% Trypsin-EDTA, gentamycin, DMEM, RPMI-1640, fetal bovine serum and bacterial growth medium. Active Fine Chemicals Limited, Dhaka, Bangladesh was the source of solvents and reagents.

### 2.2. Collection and Extraction of Acacia nilotica Flowers

*A. nilotica* was grown at the canal banks crossing the Delta, Monufia Governorate, Egypt. Flowers of *A. nilotica* were collected from three plants of the same species that were identified according to the botanical keys [31]. The plant was identified by Prof. Marei A. Hamed, Prof. of plant taxonomy. The voucher sample of *A. nilotica* has been placed in the herbal collection in the Botany and Microbiology Department, Faculty of Science, Al-Azhar University, Egypt. 

Healthy and fresh flowers were collected and washed with clean water. Then, they were dried at 50 °C until a constant weight was obtained. The ground flowers were extracted using methanol via rotary evaporator. The obtained extract was weighed and stored at 5 °C in a refrigerator until further analysis.

### 2.3. HPLC Analysis of Acacia nilotica Flower Extract

Once the extraction process finished, 5 μL of the *A. nilotica* flower extract was inserted into the HPLC apparatus (Agilent 1260 series, Agilent Technologies, Santa Clara, CA, USA). The specifics of the utilized Eclipse C18 column comprised 4.6 mm × 250 mm i.d., 5 μm and 40 °C. Mobile phase included the use of two buffers: A (0.05% trifluoroacetic acid with mili Q water) and B (0.05% trifluoroacetic acid with acetonitrile). The buffers were applied at 0.9 mL/min as a rate of flow. The serial for buffer A, which had a 20 min run for the mobile phase, was 82, 80, 60, 60, 82, 82 and 82% for 0, 0–5, 5–8, 8–12, 12–15, 15–16, 20–20 min. By using an ultraviolet (UV) detector at a wavelength of 280 nm, the contents of phenolic compounds and flavonoids were identified. The quantity of each was documented based on the injected standard compounds [32].

### 2.4. Assessment of Anti-H. pylori Activity of A. nilotica Flower Extract 

*H. pylori* was obtained from Ain Shams University Hospital, Cairo, Egypt as a tested organism to perform these experiments. The in vitro anti-*H. pylori* activities were detected using a well agar diffusion technique with the following procedures: A volume of 100 μL suspension of *H. pylori* (1.0 × 10^8^ colony forming units (CFUs)/mL) was seeded in Mueller Hinton agar amended with 10% blood and blood products and poured in plates. After solidification, a hole with a diameter of 6 mm was punched aseptically with a sterile cork borer. Then, 100 µL of the *A. nilotica* flower extract was loaded into the well. The well loaded with DMSO was utilized as the negative control, while the well loaded with clarithromycin (0.05 mg/mL) was utilized as the positive control. The inoculated plates were incubated at 37 °C with specific conditions (microaerophilic with humidity) for three days. Then, the diameter of the visualized inhibition zone was detected [33].

### 2.5. Minimal Inhibitory Concentration (MIC) Experiment

*A. nilotica* flower extract was subjected to detect its MIC against *H. pylori* via micro-dilution broth, utilizing Mueller–Hinton broth containing lysed horse blood. Different serial dilutions containing 0.98 to 1000 μg/mL of *A. nilotica* flower extract were prepared. Each appropriate dilution of *A. nilotica* flower extract (200 μL) was dispensed per well of sterile in 96-well polystyrene microtitrate plates. The inoculum of *H. pylori* from fresh culture was prepared in sterile NaCl (0.85%) to equal the standard of 1.0 McFarland turbidity. A total of 2 µL of *H. pylori* inoculum was added to each well to obtain a final dose of 3.0 × 10^6^ colony forming units)/mL. The plates were incubated under microaerophilic situations (15% CO_2_) at 35 °C for 3 days. Then, the MIC was evaluated visually, reflecting the whole growth inhibition of *H. pylori*. *H. pylori* inoculum without *A. nilotica* flower extract was used as a positive control, while *A. nilotica* flower extract without *H. pylori* inoculum was used as a negative control in each microplate [34].

### 2.6. Minimal Bactericidal Concentration (MBC) Experiment

MBC was performed via sub-culturing 100 mL of the *H. pylori* culture from each well that presented thorough inhibition of growth, from the last positive and from the growth control, onto the Mueller–Hinton agar plates (5% horse blood). The plates were incubated under microaerophilic situations (15% CO_2_) at 35 °C for 3 days. Then, the MBC was evaluated visually, reflecting complete inhibition of *H. pylori* growth by the lowest concentration of *A. nilotica* flower extract. To detect either the bactericidal or bacteriostatic agent of the *A. nilotica* flower extract, the ratios of MBC/MIC were estimated. If the MBC/MIC ratio was no higher than four times that of the MIC, the extract possessed bactericidal efficacy [35].

### 2.7. Microtiter Plate Test for Biofilm Quantification

The influence of *A. nilotica* flower extract on the formation of *H. pylori* biofilm was assessed in 96-well polystyrene flatbottom plates. Fresh trypticase soy yeast broth inoculated with *H. pylori* (250 μL containing 10^6^ CFU/mL) was transferred to each microplate and was well supported with the different levels of the sub-lethal quantity of MBC (25, 50 and 75%), which was detected in the current investigation. The plates were incubated for 48 h at 37 °C. Then, after finishing this period, the supernatant was removed. The sterile distilled water for each well was then used to remove free-floating cells. Then, the plates were dried for 30 min in the air. The formed *H. pylori* biofilm was stained by 0.1% aqueous solution of crystal violet for 15 min at 25 °C (room temperature), followed by removal of the excess of crystal violet via sterile distilled water. To completely remove the dye bound to the *H. pylori* cells, 250 μL of ethanol (95%) was added to each well as a dye-solubilized agent. Then, all wells were incubated for 15 min, and, via a microplate reader, the absorbance was measured at 570 nm [36].

(1) $$\mathrm{Inhibition}\;\mathrm{of}\;H.\;pylori\;\mathrm{biofilm}\;=1-\left(\frac{\mathrm{absorb}.\;\mathrm{sample}\;-\;\mathrm{absorb}.\;\mathrm{Blank}}{\mathrm{absorb}.\;\mathrm{control}\;-\;\mathrm{absorb}.\;\mathrm{Blank}}\right)\times 100$$

The absorbance of the media is represented with Blank, the absorbance of *H. pylori* with treatment is represented with sample, and the absorbance of *H. pylori* without any treatment is represented with control.

### 2.8. Urease Activity Inhibition Assessment 

The examined solution mixture was contained of urea (850 μL), the extract (in the range of 0 to 100 μL) and phosphate buffer (100 mM, pH 7.4) to reach the total value of 985 μL. The enzymatic reactions began with the addition of urease (15 μL). Then, they were measured via detecting the concentration of ammonia after 60 min utilizing 500 μL of solution A (composed of 0.5 g phenol and 2.5 mg of sodium nitroprusside in 50 mL of distilled H_2_O) and 500 μL of solution B (composed of 250 mg NaOH and 820 μL of NaOCl 5% in 50 mL of distilled H_2_O) for 30 min at 37 °C. The activity of uninhibited urease was selected as 100% of the control activity. The absorbance of the reaction mixture was assessed at 625 nm, and, by using the following formula, the enzymatic activity was determined:

(2) $$\mathrm{Inhibition}\;\mathrm{of}\;\mathrm{urease}\;\left(\%\right)=1-\left(\frac{T}{C}\right)\times 100$$

The absorbance of the flower extract in the existence of urease is represented as T, and the absorbance of the solvent in the existence of urease is represented as C (control) [37]. 

The concentration that induces an inhibition halfway among the minimum and maximum response of each compound (IC50) was determined to monitor the inhibition influence of different concentrations of flower extract in the assay. Inhibition activity of hydroxyurea was also evaluated as the standard compound.

### 2.9. Estimation of A. nilotica Flower Extract Antioxidant Activity via DPPH Radical Scavenging Method

*A. nilotica* flower extract was tested for its capacity to scavenge free radicals using 1,1-diphenyl-2-picryl hydrazyl (DPPH). A total of 0.1 mM DPPH solution in ethanol was prepared; then, 1 mL of this solution was added to 3 mL of *A. nilotica* flower extract in ethanol at various levels (3.9, 7.8, 15.62, 31.25, 62.5, 125, 250, 500 and 1000 g/mL). Only those extracts that were soluble in ethanol were used in this case, and different concentrations of those extracts were created using the dilution method. The mixture was vigorously shaken before being left to stand at room temperature for 30 min. Then, absorbance was determined at 517 nm (UV-VIS Milton Roy), using spectrophotometer. Ascorbic acid was used as the reference standard compound, and three copies of the experiment were run. Using a log dose inhibition curve, the sample’s IC_50_ value—the amount of *A. nilotica* flower extract necessary to inhibit 50% of the DPPH free radical—was determined [24]. The following equation was used to estimate the percentage of the DPPH scavenging outcome:

DPPH scavenging (%) = A0 − A1/A0 × 100

where A0 is the absorbance of the control reaction and A1 is the absorbance in the presence of *A. nilotica* flower extract or ascorbic acid.

### 2.10. Viability Assay for the Evaluation of the Cytotoxicity of A. nilotica Flower Extract

Cytotoxic effects of *A. nilotica* flower extract were tested against human hepatocellular carcinoma cells (HepG-2 cells) that were obtained from VACSERA Tissue Culture Unit, Egypt, and human normal melanocytes (HFB-4 cells) that were obtained from the American Type Culture Collection (ATCC, Rockville, MD, USA).

All cells were sub-cultured twice a week and kept at 37 °C in a humid environment with 5% CO_2_. Cells were seeded in a 96-well plate at a density of 1 × 10^4^ cells per well containing growth medium (100 µL). Different concentrations of *A. nilotica* flower extract were added to the fresh medium after 24 h of cell seeding. Serial two-fold dilutions of *A. nilotica* flower extract were added to reach the confluency in 96-well flat-bottomed microtiter plates (Falcon, NJ, USA) utilizing a multichannel pipette. Cells were then incubated at 37 °C in a humid environment with 5% CO_2_ for a time of 24 h. Control cells were incubated without *A. nilotica* flower extract and with or without DMSO. Three wells were utilized for each dose of the *A. nilotica* flower extract. At the end of the incubation period, the cultivated medium was aspirated. Then, 1% of the crystal violet solution (0.5% *w/v* crystal violet and 50% methanol made up to volume with ddH_2_O, followed by filtration using Whatmann No.1 filter paper) was added to each well for 30 min. The crystal violet solution was then aspirated. Then, the plates were washed using tap water to remove the excess crystal violet. An amount of 30% glacial acetic acid was added to all wells and mixed carefully. Then, the absorbance of the plates was recorded at 490 nm, and the contents were gently shaken onto the microplate reader (TECAN, Inc.), to detect the yield of viable cells. The optical density was measured with the microplate reader (SunRise, TECAN, Inc., Albany, NY, USA) to determine the viable cells percentage, which was calculated using the following formula:

[(ODt/ODc)] × 100%

where ODt is the mean optical density of wells exposed to the *A. nilotica* flower extract, and ODc iss the mean optical density of control cells [38].

The relation among living cells and *A. nilotica* flower extract concentration was plotted to find the survival curve of the cancer cell line after being exposed to *A. nilotica* flower extract. From graphic plots of the dose response curve for each concentration, the 50% inhibitory concentration (IC_50_), or the concentration required to have toxic effects in 50% of intact cells, was calculated using the Graphpad Prism software (San Diego, CA, USA). All investigations were performed in triplicate [39]. Vinblastine sulfate was applied as a positive control against cancer cells under the same experimental conditions.

### 2.11. Experimental Docking Study

Computer research has been used to determine the structural and chemical properties of this particular group of molecules that may have an effect on the apoptotic process. In this study, we looked at the positions of the inhibitor ferulic acid in the *H. pylori* (4HI0) binding site using molecular docking modeling (MOE) 2019.0102 program. The receptor structures were obtained directly from Protein Data Bank (https://www.rcsb.org/, accessed on 19 July 2022). The downloaded structures were then prepared for docking by removing all water molecules and other metal ions or ligands. The primary chain was docked, and the selected chain was then fixed and protonated utilizing the tools for structure preparation. In order to build the dummy sites that served as the binding pocket, the MOE site finder generated the active binding sites. The studied compounds were minimized and optimized for the docking process [40]. The dock scoring in MOE software was calculated using the London dG scoring function and refined using two different methods. The best five conformations that were presented in the crystal structure with a lower RMSD value were predicted by the docking process. Using the visualizing program PyMol, the complexes were examined for interactions and their 3D images were captured. Additionally, the RMSD and RMSD-refine fields were used to compare the results of pose-with-pose in the co-crystal ligand position prior to and after modification, respectively.

### 2.12. Statistical Study

All statistical studies have been expressed as mean ± standard error (SD) and the outcomes were established at least three times.

## 3. Results and Discussion

### 3.1. Phytochemical Constituents

**Figure 1:**
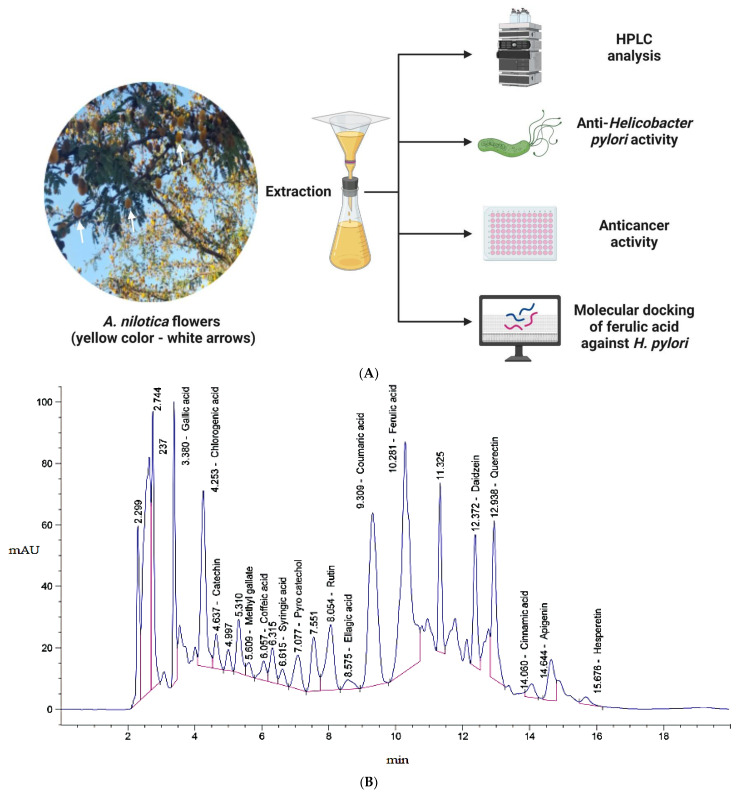
Image presenting *A. nilotica flowers* (white color), which experienced a successful extraction method and various relevant assays to detect the phytoconstituents and the anti-*H. pylori*, anti-biofilm and anticancer activities of the extracted product. This figure was created with BioRender.com, 30 March 2023 (**A**). Chromatograms of phenolic and flavonoid compounds detected in *A. nilotica* flower extract via high-performance liquid chromatography (**B**). White arrows pointed to plant flowers (yellow color).

**Figure 2:**
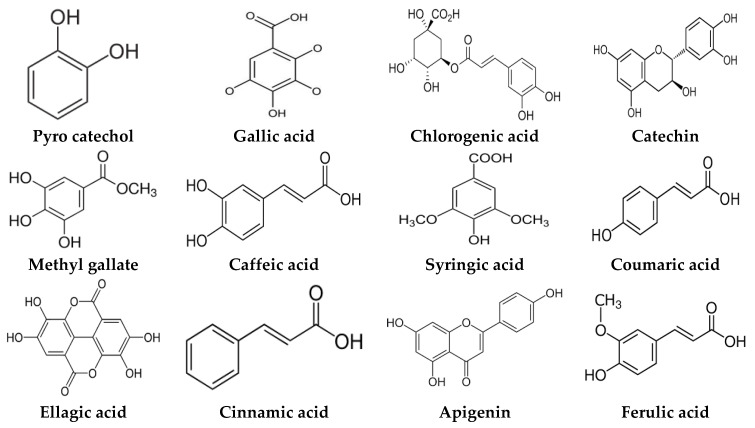

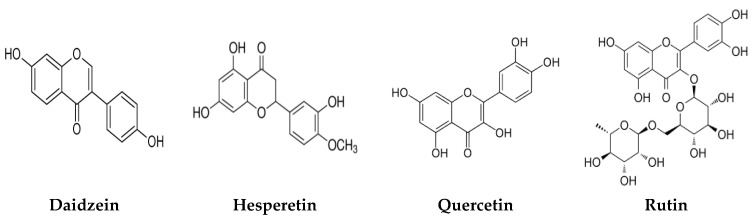
Chemical formula detected in *A. nilotica* flower extract.

*A. nilotica* flower (Figure 1A) extract was subjected to HPLC analysis, and the incidence of different phenolic and flavonoid constituents with different retention times, concentrations, area and area % (Figure 1B and Table 1), and chemical formulas was determined (Figure 2). Three compounds were recognized in the extract with great quantity: 5451.04, 4572.26 and 3733.37 µg/mL for ferulic acid, chlorogenic acid and quercetin, respectively. Other detected compounds such as rutin (2393.13 µg/mL) and gallic acid (2116.77 µg/mL) were reported with moderate concentrations. Cinnamic acid (69.72 µg/mL), hesperetin (121.39 µg/mL) and methyl gallate (140.45 µg/mL) were distinguished with the lowest quantity. Moreover, other compounds in the extract, including catechin, caffeic acid, syringic acid, pyro catechol, ellagic acid, apigenin daidzein and coumaric acid were detected. Unfortunately, eight unidentified compounds were present in the extract due to the defect of the standard compounds in the HPLC system. On the other hand, some compounds including vanillin, naringenin and kaempferol were not found in the extract. There are differences in the detected phenolic and flavonoid compounds according to the organ, the geographical location of the cultivation area, the extraction methods and the nutritional and climatic condition of the plant. Sayed et al. [41] recorded the presence of catechin, catechin 7-O-gallate, gallic acid, naringenin 7-O-β-glucopyranoside and quercetin in *A. nilotica* flower extract. The major detected compounds in *A. nilotica* ripe fruits were gallic acid methyl ester, followed by catechin and catechin-7-gallate [42]. In another recent study, Kaur et al. [4] studied the phytoconstituents of *A. nilotica* bark extract via HPLC, showing the existence of numerous phenolic constituents such as ferulic acid, myricetin, kaempferol, rutin, quercetin, gallic acid, betulin, catechin and epicatechin. The extracted *A. nilotica* flowers with aqueous methanol reflected the presence of gallic acid, catechin, 7-Galloyl catechin, quercetin 3-O-rhanmnopyranosyl (1→6) glucopyranoside, quercetin-3-O-α-L-rhamnopyranoside, quercetin 3-O-β-glucopyranoside and quercetin [29]. Several studies have focused on the biological activities of the natural phenolic and flavonoid molecules in the fields of pharmaceutics and nutrition. Most of the detected compounds in the extract have been shown to have anticancer, antimicrobial, antioxidant, anti-inflammatory and anti-diabetic properties, but these were illustrated in studies that were conducted on other plants. *Yersinia enterocolitica* was inhibited by chlorogenic acid [43]. Quercetin exhibited therapeutic potential for treating inflammation, Alzheimer, diabetes and cancer, showing some antioxidant activities [44,45]. Rutin was also reported in inflammation and thrombosis treatments [46].

### 3.2. Anti-Helicobacter Pylori Activity of A. nilotica Flower Extract

**Figure 3:**
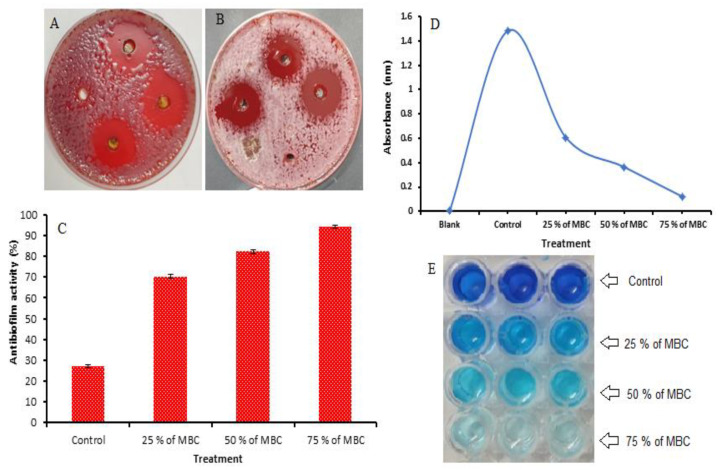
Inhibitory activity of *A. nilotica* flower extract (**A**). Clarithromycin as a positive control against *H. pylori*. Wells without inhibition zone are considered as negative control (DMSO) (**B**). Anti-biofilm of *A. nilotica* flower extract against *H. Pylori* (**C**). Biofilm analysis of *A. nilotica* flower extract against *H. Pylori* at different concentration of MBC (**D**). Microtiter plate showing color changes as an indicator of decreased *H. Pylori* viability at media+ *H. Pylori* (Control), 25% of MBC of flower extract, 50% of MBC of flower extract and 75% of MBC of flower extract (**E**).

Inhibitory activity was attributed to *A. nilotica* flower extract against *H. pylori* with an inhibition zone of 31 mm compared to the clarithromycin of 21.67 mm as a positive control (Table 2 and Figure 3A,B). Clarithromycin was selected because it is a broad-spectrum antibiotic that is utilized for the treatment of a wide variety of infections caused by bacteria, including *H. pylori* infection. Clarithromycin inhibits bacteria via binding to the subunit 50S, leading to the inhibition of protein synthesis. Excellent MIC and MBC were recorded in the *A. nilotica* flower extract with 7.8 µg/mL and 15.62 µg/mL, respectively; however, both the MIC and the MBC of the positive control were 31.25 µg/mL. Via the calculation of the MBC/MIC index, it is clear that the *A. nilotica* flower extract is determined to be a bactericidal agent due to MBC/MIC values that were less (2.0) than the value of the MIC in the positive control, not exceeding four times that of the positive control MIC (Table 2). Amin et al. [25] reported that the MIC of *Helicobacter pylori* was found to be 8 μg/mL with the treatment of *A. nilotica* extract. According to Auwal et al. [47], a pod crude extract of *A. nilotica* exhibited a killing effect against *Staphylococcus aureus* (MIC, 200 mg/mL; MBC, 200 mg/mL), *Streptococcus pyogenes* (MIC, 25 mg/mL; MBC, 100 mg/mL), *Bacillus subtilis* (MIC, 12.5 mg/mL; MBC, 25 mg/mL), *Corynebacterium pyogenes* (MIC, 12.5 mg/mL; MBC, 200 mg/mL), *Klebsiella pneumoniae* (MIC, 12.5 mg/mL; MBC, 25 mg/mL) and *Candida albicans* (MIC, 25 mg/mL; MBC, 25 mg/mL). This investigation showed the importance of *A. nilotica* flower extract as an anti-*H. pylori* agent and its applications in the treatment of *H. pylori* ulcers. The inhibitory mechanism of phenolic and flavonoid compounds against pathogenic microorganisms has been elucidated in a number of studies [48]. Asha et al. [49] reported that dihydrofolate reductase, DNA gyrase and protein synthesis in *H. pylori* were inhibited by a flavonoid-rich plant extract. One of the virulence factors, urease of *H. pylori*, was also inhibited by definite phenolic compounds [50]. Campos et al. [51] studied the antibacterial activity mechanism of ferulic, *p*-coumaric and caffeic acids and mentioned the capability of these compounds to interfere with bacterial membrane integrity. Flavonoids such as naringenin, taxifolin and eriodictyol can also interact with several enzymes responsible for the creation of bacterial cell membrane precursors and those responsible for the elongation of fatty acid cycle in *Enterococcus faecalis* [52]. In another study, methicillin-resistant *Staphylococcus aureus* peptidoglycan and cell wall biosynthesis were damaged as a result of the exposure to sophoraflavanone B [53].

Moreover, anti-biofilm activity (%) with the increment of the MBC of the *A. nilotica* flower extract against *H. pylori* showed 70.38, 82.29 and 94.22% using 25%, 50% and 75% of MBC, respectively. In the control, the anti-biofilm activity was 27.12% (Figure 3C). A plot of the absorbance versus the levels of MBC utilized is visualized (Figure 3D). The color change in the stained biofilm in the microtiter plate was dependent on the viability of the biofilm (Figure 3E). Several bacterial species, such as *Bacillus subtilis*, *B. cereus*, *B. megaterium*, *S. aureus*, *B. aryabhattai*, *Pseudomonas putida*, *Serratia marcescens*, *Escherichia coli* and *K. pneumoniae*, were impacted by *A. nilotica* leaf extracts [30].

In this study, anti-*Helicobacter pylori* by a *A. nilotica* flower extract was assessed and the possible inhibitory influence on its connected urease was also documented. Urease inhibition (%) increased with the increased concentration of the flower extract and reached 76.6% at 1000 µg/mL. The recorded IC_50_ was 67.4 µg/mL (Table 3). Zhou et al. [54] reported the inhibition of *H*. *pylori* urease by almatine from *Coptis chinensis*, in a dose-dependent manner, with IC_50_ values of 0.53 ± 0.01 mM, in comparison to acetohydroxamic acid, a well-known urease inhibitor (0.07 ± 0.01 mM). Gastric urease permits the *H. pylori* to inhibit the acidic stomach and functions as a biosign for the occurrence of *H. pylori*. One of the virulence factors of *H. pylori* was associated with urease to create the environment’s alkaline by transforming urea into ammonia and CO_2_ [55].

### 3.3. Molecular Docking of Ferulic Acid with 4HI0 Protein of H. pylori

**Figure 4:**
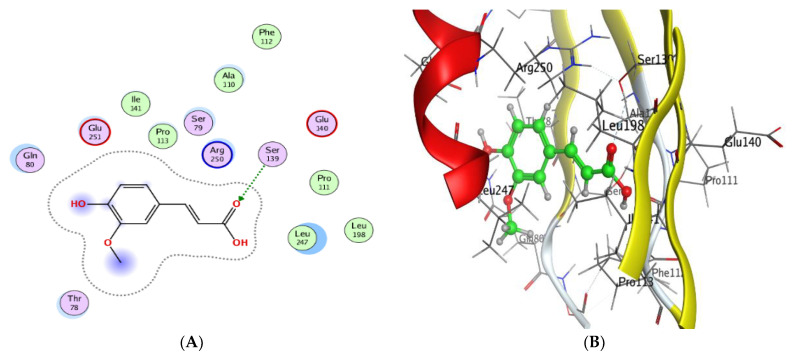

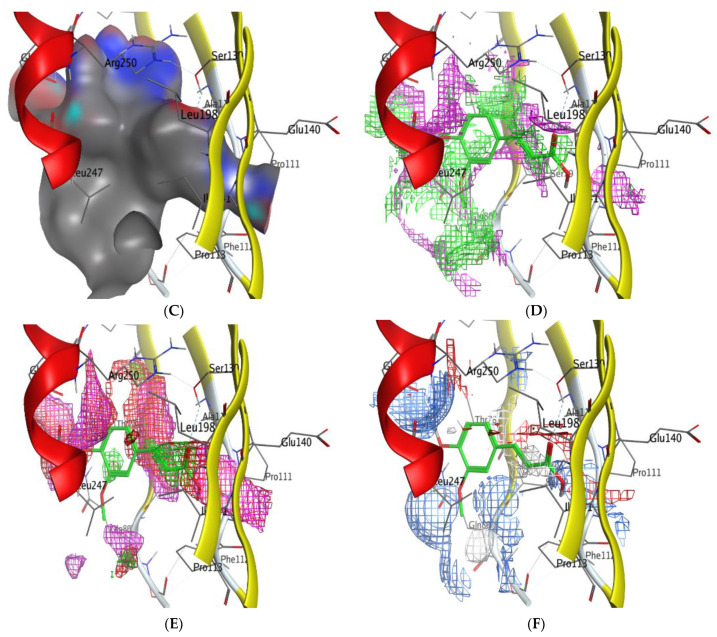
Molecular docking process of ferulic acid with 4HI0. The interaction between ferulic acid and active sites of 4HI0 protein (**A**). The most likely binding conformation of ferulic acid and the corresponding intermolecular interactions are identified (**B**). Molecular surface of ferulic acid with 4HI0 (**C**). The contact preference of ferulic acid with 4HI0 (**D**). Interaction potential of ferulic acid with 4HI0 (**E**) and the electrostatic map of ferulic acid with 4HI0 (**F**).

Ferulic acid, which was the highest detected compound in the *A. nilotica* flower extract, was investigated against 4HI0 protein of *H. pylori*. The goal of molecular docking, an optimization issue, is to identify the ligand binding mode that has the lowest potential energy. The target binding site’s coordinate space was sampled during docking, and each potential ligand, which had been posed within that site, was scored. The ligand posture with the highest score was then used to forecast the binding mode for that chemical. A 2D molecular structure of ferulic acid has been assigned to the MOE (Molecular Operating Environment), which was designed to visualize the active sites of the complexes (protein–ligand) (Figure 4A–F). The ligand was arranged and rendered using an improved version of the 2D algorithm layout representation, and the protein residues were arranged around it to indicate spatial proximity bonds. The obtained results revealed that ferulic acid was a good inhibitor for the (4HI0) enzyme to slow the progression of *H. pylori*. Ferulic acid has been found to interact with the residue’s SER 139 active site via the O 29 atom and had a low energy score (−5.58 Kcal/mol), which was important for their biological activity (Table 4 and Table 5). The increased negative score of the free binding energy in the current study confirmed the mechanism of bacterial growth inhibition by ferulic acid. This result was in agreement with recent studies [24,56].

The molecular docking of natural constituents such as chlorogenic acid and pyrocatechol was reported with the *H. pylori* (4HI0) protein in a recent study [24], which supported the efficacy of these constituents against *H. pylori* growth. Other investigations also documented the application of the molecular docking of natural ingredient interactions with the *E. coli* 7C7N protein (value of energy −6.04 kcal mol^−1^) [40] and *Proteus vulgaris* [2].

### 3.4. Antioxidant Activity A. nilotica Flower Extract

The *A. nilotica* flower extract presented appropriate antioxidant activity depending on the extract concentration. As the concentration increased, the antioxidant properties of the extract increased. For instance, at 15.63, 62.50, 250 and 1000 µg/mL, the DPPH scavenging percentage was 42.3, 52.6, 65.5 and 80.6%, respectively, with an IC_50_ of 36.74 µg/mL (Table 6). All findings of the antioxidant properties were judged using standard drug ascorbic acid, which reflected an IC_50_ of 4.08 µg/mL. The promising antioxidant activity was associated with the *A. nilotica* flower extract content of the phenolic and flavonoids constituents. In the current study, the antioxidant activity of *A. nilotica* flower extract was better than using other parts of the plant, as shown in comparison to some previous studies. For example, Subhaswaraj et al. [57] studied the antioxidant activity *A. nilotica* leaf extract and showed an IC_50_ of 75.157 µg/mL. Polyphenols from the extract of *A. nilotica* ripe fruits demonstrated an IC_50_ of 41.91 µg/mL [42]. Another study showed that catechins, a constituent of the *A. nilotica* flowers, could show antioxidative potential [10].

### 3.5. Anticancer of A. nilotica Flower Extract

The aerial part extracts of *Acacia hamulosa*, *A. salicina*, *A. laeta* and *A. tortilis* displayed a moderate effect against cancer proliferation in kidney, breast and liver cells [19]. In the current study, the flower extract of *A. nilotica* exhibited antitumor potential against HepG-2 cells with different levels dependent on the concentration of the extract. A promising result was obtained at a high concentration, in which the inhibitory activity was 91.26% and 96.73% at 500 µg/mL of the flower extract and Vinblastine sulfate against HepG-2 cells, respectively (Table 7). The IC_50_ was 176.15 µg/mL against HepG-2 cells, while the IC_50_ was 395.30 µg/mL against HFB-4 cells, which reflects its safe utilization against cancer cells. However, several studies reported the anticancer properties of natural ingredients, but, after reviewing these studies, it appeared that anticancer potential depended on chemical structure, the type of the ingredients and concentration and genomes of tumor cells [29,58]. These remarks coincided with other reports using different parts of *A. nilotica* [59]. Different IC_50_ values of *A. catechu* fruit extract ranging from 9.7 to 42.8 µg/mL were reported against human cancer cell lines such as those of MCF-7, A549 and THP-1 cells [28]. In a recent study, the anticancer effects of *A. nilotica* with IC_50_ values of 59.3 µg/mL and 96.9 µg/mL were observed against A549 and MCF-7 cells, respectively, while less anticancer effects were observed against THP-1 cells with an IC_50_ of more than 100 µg/mL [29]. The influence of *A. nilotica* flower extract on normal cells was parallel with Revathi et al. [30], in which the *A. nilotica* leaf extracts documented the anticancer activity of the extracts against Hep-2 (cervical cancer) and MDA-MB-231 (breast cancer) and showed sparing effects on normal Vero cells. As mentioned from the HPLC analysis, ferulic acid was the main component detected in the *A. nilotica* flower extract. The mechanisms of ferulic acid cytotoxicity against cancer cells was studied, from which autophagy and apoptosis of HepG2 were observed with the disruption of nucleoli, morphological alterations, cell proliferation decline, mitochondrial membrane potential decrement, and an increased % of cells in the S phase [60]. In a review report [61], cancer cells treated with ferulic acid characterized with DNA fragmentation lowered the rate of protein kinase phosphorylation. Sundarraj et al. [62] reported that the MCF-7 cell cycle at the G2/M phase was blocked as a result of the exposure to a leaf extract of A. nilotica. Another species, namely, Acacia catechu, also arrested the human colon cancer cell cycle at this phase and showed a decline in the potential mitochondrial membrane [63].

## 4. Conclusions

This study offered additional scientific support to the utilization of *A. nilotica* flower extract to treat *H*. *pylori*-related gastrointestinal infections. *A. nilotica* flower extract might be a helpful treatment for peptic and gastritis ulcers induced by *H*. *pylori* infection and other urease-related illnesses. The results of this study suggest that the *A. nilotica* flower extract, due to its considerable antioxidant potential, might play a role in protection via the avoidance of lipid peroxidation generated by free radicals. Furthermore, based on the obtained findings, further studies must be conducted to document the biological effects of *A. nilotica* flower extract in vivo, and detailed mechanistic studies are required to validate these biological applications.

## Figures and Tables

**Table 1:** Flavonoid and phenolic compounds detected in *A. nilotica* flower extract.

Compound	Retention Time	Area	Area (%)	Concentration (µg/mL)
Unknown	2.299	337.98	4.16	Undetected
Unknown	2.637	1081.92	13.31	Undetected
Unknown	2.744	488.57	6.01	Undetected
Gallic acid	3.38	420.91	5.18	2116.77
Chlorogenic acid	4.253	574.10	7.06	4572.26
Catechin	4.637	106.39	1.31	1524.96
Unknown	4.997	58.61	0.72	Undetected
Unknown	5.310	174.94	2.15	Undetected
Methyl gallate	5.609	44.12	0.54	140.45
Caffeic acid	6.057	73.77	0.91	333.93
Unknown	6.315	121.44	1.49	Undetected
Syringic acid	6.615	54.70	0.67	222.45
Pyro catechol	7.077	158.71	1.95	1303.73
Unknown	7.551	217.63	2.68	Undetected
Rutin	8.054	349.54	4.30	2393.13
Ellagic acid	8.575	57.27	0.70	683.49
Coumaric acid	9.309	969.16	11.92	1715.35
Vanillin	9.808	0.00	0.00	0.00
Ferulic acid	10.281	1381.53	16.99	5451.04
Naringenin	10.494	0.00	0.00	0.00
Unknown	11.325	346.87	4.27	Undetected
Daidzein	12.372	333.41	4.10	1190.40
Quercetin	12.938	491.94	6.05	3733.37
Cinnamic acid	14.060	65.05	0.80	69.72
Apigenin	14.644	182.79	2.25	796.88
Kaempferol	15.061	0.00	0.00	0.00
Hesperetin	15.676	39.19	0.48	121.39

**Table 2:** Inhibitory activity, MIC and MBC of *A. nilotica* flower extract against *H. pylori*.

Treatment	Mean Inhibitions Zones (mm)	MIC (µg/mL)	MBC (µg/mL)	MBC/MIC Index
Extract	31.00 ± 1.00	7.8	15.62	2.0
Control	21.67 ± 1.53	31.25	31.25	1.0

**Table 3:** Effect of different concentrations of flower extract on urease activity.

Concentration (µg/mL)	Flower Extract		
	OD Mean	Urease Inhibition %	±SD
1000	0.172	76.6	0.003
500	0.236	67.9	0.004
250	0.276	62.4	0.006
125	0.338	54.0	0.004
62.5	0.375	48.9	0.004
31.25	0.422	42.6	0.003
15.625	0.458	37.6	0.002
7.8125	0.512	30.3	0.004
3.9	0.553	24.8	0.002
1.95	0.621	15.5	0.002
0.0	0.735	0.0	0.026
IC_50_	67.4 µg/mL		

**Table 4:** Docking scores and energies of ferulic acid with crystal structure of *H. pylori* 4HI0.

Mol (Five Poses of Ferulic Acid)	rseq	mseq	S	rmsd_refine	E_conf	E_place	E_score1	E_refine	E_score2
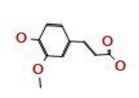	1	1	−5.58	3.35	−42.86	−61.32	−11.22	−27.30	−5.58
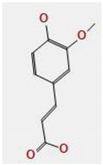	1	1	−5.35	1.81	−40.17	−64.29	−11.15	−29.20	−5.35
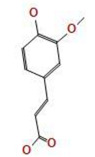	1	1	−5.31	2.06	−41.95	−63.97	−9.95	−28.10	−5.31
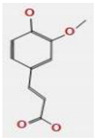	1	1	−5.29	1.73	−41.47	−67.91	−10.93	−27.96	−5.29
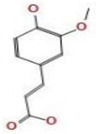	1	1	−5.24	2.00	−40.25	−74.10	−9.96	−28.98	−5.24

S, the final score (score of the last stage that was not set to none); rmsd, the root-mean-square deviation of the pose, in Å, from the original ligand; rmsd_refine, the root-mean-square deviation among the pose before refinement and the pose after refinement; E_conf, the energy of the conformer; E_place, the score from the placement stage; E_score 1 and E_score 2, the score from rescoring stages 1 and 2, respectively; E_refine, the score from the refinement stage, which is determined to be the totality of the solvation energies and van der Waals electrostatics, according to the generalized Born solvation model.

**Table 5:** Interaction of ferulic acid with crystal structure of *H. pylori* 4HI0.

Mol	Ligand	Receptor	Interaction	Distance	E (kcal/mol)
Ferulic acid	O 20	OG SER 139 (B)	H-acceptor	3.02	−0.7

**Table 6:** DPPH scavenging % of *A. nilotica* flower extract and ascorbic acid.

Concentration (µg/mL)	Ascorbic Acid			Flower Extract		
	OD Mean	DPPH Scavenging %	SD	OD Mean	DPPH Scavenging %	SD
1000	0.049	97.0	0.002	0.322	80.6	0.007
500	0.091	94.5	0.004	0.437	73.6	0.007
250	0.122	92.7	0.005	0.572	65.5	0.004
125	0.225	86.4	0.006	0.689	58.4	0.008
62.50	0.365	78.0	0.006	0.787	52.6	0.005
31.25	0.478	71.2	0.004	0.866	47.8	0.009
15.63	0.593	64.2	0.005	0.956	42.3	0.010
7.81	0.725	56.3	0.003	1.054	36.4	0.009
3.90	0.898	45.8	0.002	1.130	31.8	0.004
1.95	0.966	41.7	0.007	1.215	26.7	0.003
0	1.658	0.0	0.004	1.658	0.0	0.004
IC_50_	4.08 µg/mL			36.74 µg/mL		

**Table 7:** Cytotoxic activities of *A. nilotica* flower extract and vinblastine sulfate.

Concentration (µg/mL)	Flower Extract				Vinblastine Sulfate	
	HepG-2 Cells		HFB-4 Cells		HepG-2 Cells	
	Viability %	Inhibitory %	Viability %	Inhibitory %	Viability %	Inhibitory %
500	8.74	91.26 ± 0.62	32.65	67.35 ± 2.31	3.27	96.73 ± 1.48
250	30.96	69.04 ± 1.48	74.08	25.92 ± 2.14	5.89	94.11 ± 1.30
125	63.19	36.81 ± 2.35	91.47	8.53 ± 0.69	10.92	89.08 ± 1.25
62.5	84.23	15.77 ± 1.09	98.16	1.84 ± 0.48	14.36	85.64 ± 0.31
31.25	97.58	2.42 ± 0.64	100	0.0	19.24	80.76 ± 0.48
15.6	100	0.0	100	0.0	26.85	73.15 ± 1.25
7.8	100	0.0	100	0.0	34.19	65.81 ± 0.50
3.9	100	0.0	100	0.0	45.06	54.94 ± 1.33
2	100	0.0	100	0.0	54.28	45.72 ± 1.42
1	100	0.0	100	0.0	60.94	39.06 ± 0.45
0	100	0.0	100	0.0	100	0.0
IC_50_	176.15 ± 5.08 µg/mL		395.30 ± 11.49 µg/mL		2.93± 0.33 µg/mL	

## Data Availability

Not applicable.
